# Education on tick bite and Lyme borreliosis prevention, aimed at schoolchildren in the Netherlands: comparing the effects of an online educational video game versus a leaflet or no intervention

**DOI:** 10.1186/s12889-016-3811-5

**Published:** 2016-11-16

**Authors:** D. J. M. A. Beaujean, F. Gassner, A Wong, J. E. Steenbergen, R. Crutzen, D. Ruwaard

**Affiliations:** 1 National Institute for Public Health and the Environment, Centre for Infectious Disease Control, P.O. Box 1, 3720 BA Bilthoven, The Netherlands; 2 Department of Statistics, Informatics and Mathematical Modeling, National Institute for Public Health and the Environment, P.O. Box 1, 3720 BA Bilthoven, The Netherlands; 3 Department of Infectious Diseases, Leiden University Medical Center, P.O. Box 9600, 2300 Leiden, The Netherlands; 4 Department of Health Promotion, Faculty of Health, Medicine and Life Sciences, CAPHRI School for Public Health and Primary Care, Maastricht University, P.O. Box 616, 6200 Maastricht, MD The Netherlands; 5 Department of Health Services Research, Faculty of Health, Medicine and Life Sciences, CAPHRI School for Public Health and Primary Care, Maastricht University, P.O. Box 616, 6200 Maastricht, MD The Netherlands

**Keywords:** Educational video game, Leaflet, Ticks, Tick bites, Schoolchildren, Lyme borreliosis, Lyme disease, Prevention, Tick check, Knowledge, Perception

## Abstract

**Background:**

Lyme disease or Lyme borreliosis (LB) is the most common tick-borne disease both in the United States and Europe. Children, in particular, are at high risk of contracting LB. Since child-specific educational tools on ticks, tick bites and LB are lacking, we developed an online educational video game. In this study, we compared the effectiveness of an online educational video game versus a newly developed leaflet aimed to improve prevention of tick bites and LB among Dutch schoolchildren.

**Methods:**

A total of 887 children, aged 9–13 years and attending the two final years of primary schooling, were recruited from 25 primary schools in June and July 2012. They were assigned through cluster randomization to one of three intervention groups: ‘game’ (22.4%), ‘leaflet’ (35.6%) or ‘control’ (41.9%). Prior to and directly following intervention, the children were asked to complete a short questionnaire. The main outcome measures were knowledge, perception (perceived susceptibility and importance) and preventive behavior in relation to tick bites and LB. Generalized linear mixed models were used to analyze the data.

**Results:**

In the game group, the leaflet group and the control group, knowledge about ticks and tick bites improved significantly. The game was also an effective tool for improving preventive behavior; the frequency of checking for ticks increased significantly. However, there were no significant differences in knowledge improvement between the interventions. The game outperformed the leaflet in terms of improving preventive behavior, whereas the frequency of tick checks increased significantly. But this frequency didn’t increase more than in the control group.

**Conclusions:**

The positive knowledge effects observed in the control group suggests the presence of a mere measurement effect related to completion of the questionnaire. The game did not outperform the leaflet or control group on all outcome measures. Therefore, the game may be of value as a complementary role, in addition to other media, in child-specific public health education programs on ticks and LB.

This trial was retrospectively registered on October 21, 2016 (trial registration number: ISRCTN15142369).

**Electronic supplementary material:**

The online version of this article (doi:10.1186/s12889-016-3811-5) contains supplementary material, which is available to authorized users.

## Background

Lyme borreliosis (LB), also known as Lyme disease, is caused by different *Borrelia* species from the *Borrelia burgdorferi* sensu lato group, which in Europe is transmitted by the tick *Ixodes ricinus*. The most common clinical manifestation of LB is erythema migrans (EM), a characteristic rash expanding from the site of the tick bite, which may appear some days to weeks following infection, and is sometimes accompanied by systemic flu-like symptoms. Late and more serious LB can present as a multisystemic disease with skin, neurological, cardiac and musculoskeletal manifestations (such as arthritis) [1].

Lyme disease or LB is the most commonly reported tick-borne disease in both the United States (US) and Europe. Since recording of LB began in 1991 by the Institution of Nationally Notifiable Diseases Surveillance System in the US, there has been a consistent increase in the number of annually reported cases. In 2014, it was the fifth most common notifiable disease in the US. In that year, over 25,000 confirmed cases and 8,000 probable cases were reported to the Centers for Disease Control and Prevention (CDC), although recent data suggest that an estimated 300,000 people in the US are diagnosed with LB annually [1]. Despite substantial efforts to control LB in recent decades, it is still the most prevalent tick-borne disease in the temperate regions of the northern hemisphere. Incidence rates in Europe are lower in northern Europe compared to the southern parts of Central Europe, ranging from less than one case to around 350 per 100,000 population [2]. These numbers are likely an underestimate since case reporting is inconsistent and many infections go undiagnosed [3, 4].

In the Netherlands, a repeated retrospective study among general practitioners (GPs) has shown a continuing and strong increase in consultations for tick bites between 1994 (33,000) and 2009 (93,000) [5]. In 2007, more than one million people in the Netherlands (8% of the total population) suffered a tick bite. This poses a progressive threat to public health. Rizolli, Stanek and Bacon found that, in Europe, children aged 5–14 years are at the highest risk of contracting LB since their daily life and play routines make them more prone to tick bites [2, 6, 7]. The incidence of tick bites in Dutch children aged 10–19 years was 10,866 per 100,000 population, in the most recent retrospective cross-sectional survey in 2007. This incidence exceeded that year’s mean incidence of 7,198 tick bites per 100,000 inhabitants among all ages [5]. The Dutch data accords with findings of a LB seroprevalence survey conducted among German children, indicating children as a distinct and vulnerable risk group [8].

Strategies to prevent tick bites and LB have targeted the environment and vertebrate hosts of deer ticks. Other strategies include the avoidance of tick-infested areas, the use of protective clothing (i.e., wearing long-sleeved shirts and trousers, aimed to decrease the area of exposed skin), routine checks of one’s body for ticks, and the use of tick repellents on either the skin or clothing. Although numerous prevention strategies are available, which differ in terms of costs, acceptability and effectiveness, uptake of behavioral strategies has been universally poor. Research in endemic areas has demonstrated that despite adequate knowledge about its symptoms and transmission, many people do not actively attempt to reduce their risk of infection [9–11]. Wearing protective clothing and using insect repellent skin products are less accepted measures, while checking the body and removing ticks are deemed to be more acceptable by the general public in the Netherlands [9]. Corapi concluded in his study that new prevention strategies should aim to increase people's confidence in their ability to carry out preventive behaviors, raise awareness of desirable outcomes, and aid in the realization that the necessary skills and resources are available for preventive measures to be taken [10]. Earlier we found that only 18% of the children were routinely checked for ticks by their parents [12]. De Vries et al. concluded, in their analysis of the determinants of tick inspection by parents, that education programs should clearly indicate how the need for tick inspection should be communicated to children [13]. Therefore, there is an unmet need for educational tools on ticks, tick bites and LB prevention aimed specifically at children. Since online video games are popular among children, both boys and girls, this medium is a potential medium to reach children with information about tick bites and LB [14]. Almost all children between 6 and 12 years play online video games [15]. Previous studies have demonstrated that educational video games improve young people’s knowledge, skills, attitudes and behaviors in relation to health [16–18]. Educational video games are experiential, creating a platform for active learning [19]. Rather than a didactic presentation found in e.g. a leaflet, which requires memorization or assimilation of out-of-context facts, educational video games promote ‘situated learning’ in which players discover and learn through exploration and experimentation [20, 21]. By helping players see ‘the big picture’, educational video games may help players make meaningful connections between events, for instance: playing in nature, incur a tick bite, failing to perform a tick check and subsequently becoming ill, versus always performing a tick check after playing in nature and staying healthy. This may increase the likelihood that knowledge and skills attained in the game world will be retained and applied in the real world [19].

The Dutch National Institute for Public Health and the Environment (RIVM) has produced several educational tools on tick bites and LB directed at adults (including a website, a leaflet and a movie), but no tools aimed specifically at children. As part of this study we developed, for the first time, an online educational video game available on the website www.teekcontrol.nl. We investigated the effects of this game on knowledge, perception and behavior in relation to ticks and tick bites. We compared the effectiveness of the game with that of a leaflet, containing the same take-home messages, and a control group in which children received no additional information.

## Methods

### Study design & subjects

In a pre-intervention study conducted in February-March 2012 (*t1*), municipal health services (MHS) in the Netherlands contacted primary schools to recruit children (9–13 years, mixed gender and ethnicity) by telephone, e-mail, or advertisement in MHS newsletters. In total, 1,447 children from 40 schools participated in this study by completing a specifically developed and pretested compact paper questionnaire [12]. This pre-intervention study aimed to examine the knowledge, perceived threat, and perceived importance of preventive behaviour in relation to tick bites and their potential consequences. Seventy percent of the children had a good knowledge of ticks and the potential consequences of tick bites. Knowing persons who personally got ill after a tick-bite was associated with a good knowledge score and leads to higher susceptibility and better appreciation of the need for body checks. Perceived severity was associated with a good knowledge score and with knowing persons who got ill after a tick-bite. Based on the results of this study, we concluded that it seemed to be useful to focus in future health education regarding ticks and tick-borne diseases on children besides parents.

In June and July 2012 (*t2*), study participants were recruited by contacting children of the two final grades (grade 7 and 8) from the 40 primary schools who had participated in the pre-intervention survey (Table 1). Twenty-five out of the 40 schools involved in the pre-intervention study at *t1*, participated again at *t2*. We have no information about the reasons for those schools that were lost to follow-up. Children of the 25 schools were randomly assigned per class to either the intervention groups ‘game’ or ‘leaflet’, or to the control group. To ensure that schools were spread out evenly across the Netherlands a cluster randomization sampling design was used. The Netherlands was divided into regions, which allowed sampling of schools per region and then random selection of classes per school.

**Table 1:** Study design

Date	Total number		Total number included in analysis			
	Schools	Children	Schools	Children		
*t1* ^*a*^	40	1447	25	981		
February-March 2012 Questionnaire 1				*Game*	*Leaflet*	*Control*
				*group* ^*b*^	*group* ^*b*^	*group* ^*b*^
				254	328	399
*t2*	25	887	25	887		
June-July 2012 Questionnaire 2				*Game*	*Leaflet*	*Control*
				199 (78.3%)	316 (96.3%)	*372* (93.2%)

^a^Pre-intervention study [12]

^*b*^Only *t1* participants whose school also participated at *t2* were included for analysis

Children either: played the game individually on a personal computer (game group); read a leaflet containing similar information as the game (leaflet group); or received no information (control group). Directly following the intervention (*t2*), the children completed questionnaire 2 (Additional file 1: Appendix 1). Ninety percent of the children (887/981) who participated in the pre-intervention study at *t1*, participated again at *t2*. Absence due to illness was the most important reason for loss to follow-up. Since the children completed the questionnaire anonymously at *t1*, they were grouped per class to enable comparative analysis of *t1* and *t2*. Questionnaire 2 at *t2* included the same questions as the questionnaire at *t1* (pre-intervention study) complemented with questions about the appreciation of the intervention for the intervention groups.

### Intervention materials

We developed an online educational video game www.teekcontrol.nl, based on the results of the pre-intervention study [12]. The scenario of the online educational video game www.teekcontrol.nl is that the player drives around in one’s own neighborhood (selected by entering their postal code) and is then faced with different fictive risky situations for tick bites. An example of such a risky situation is children playing in nature and picking flowers in the bushes. The player has to chase tick bite cases across a map as quickly as possible, and while doing so emit warnings that encourage people to check for ticks. The faster the warning is emitted, the more points they earn. It is possible to play the video game individually, or in a league to become the best tick controller in town. At the end of the game, the children obtain their total score. They get the opportunity to inform their own parents (or responsible adult / guardian / carer) of their game results by sending an automatically generated e-mail about the score. This e-mail also includes information on ticks and LB for the parents. In addition, it is possible to share an automatically generated message via Facebook, Twitter and Hyves (the latter was a now defunct Dutch social network site), indicating you have played the game.

The leaflet was also specifically developed by RIVM for this study and explains to children in simple language and clear pictures what ticks look like, where and how they live, where they bite on the body and when it is important for them to ask parents to (help self-)check for ticks (Additional file 2: Appendix 2). The core lessons of the leaflet and the game are the same, only the mode of delivery differed.

In a similar vein to the pre-intervention study [12], the game and the leaflet focus on determinants of preventive behavior in accordance with the Protection Motivation Theory [22, 23]. This theory posits that a ‘threat appraisal’ is formed by an individual based on the perceived likelihood of a particular event (denominated here as ‘perceived susceptibility’) and its perceived severity. In the game we tried to simulate a threat appraisal by allowing the player to drive in one’s own neighborhood and making them face different risky situations with potential exposure to ticks. During the game, new risky situations appear in the game-field. The specific characteristics of these risky situations are also described point by point next to the game-field, thereby allowing players to recognize these situations in reality too. This is the first objective of the game: to teach children to identify risky situations with potential exposure to ticks.

The second objective is to teach children the right coping appraisal. From the pre-intervention study at *t1* it is known that only 18% of the children were, at that time point, routinely checked for ticks by their parents after ‘high-risk outings’. In the game, we tried to influence this coping appraisal by stimulating the players to alert others about the need to perform a tick check as soon as possible after a visit to an area with a high tick concentration. The faster the player reaches a tick bite situation and then emits ‘the tick check alert’, the more points that are earned. Herewith, the right coping appraisal (i.e., request a tick check from a parent after having been in an area with a high potential for exposure to ticks) is rewarded in the game [24]. In the leaflet these two lessons are elaborated in text and pictures (Additional file 2: Appendix 2).

### Questionnaires

The developed questionnaires were pretested among a sample of primary schoolchildren similar to our target group, and amended slightly as a result. Since our subjects are primary schoolchildren, the questions have to be limited in number and easily understood by children. Answers were presented as two or three options, text was limited to short sentences, and images were used when possible. We included the following constructs: knowledge (assessed by asking 7 questions on tick ecology, basic prevention, and tick bites); perceived susceptibility (asking the respondents whether they think they could personally become ill after a tick bite); an additional proxy for perceived susceptibility (asking whether the respondent personally knows someone who became ill after a tick bite); perceived importance of preventive behavior as a proxy for response efficacy (asking whether the respondent thinks tick-checks are important), and actual preventive behavior (asking for the frequency of tick checks performed by the respondent’s parent(s)).

Furthermore, children were asked whether they had been given previous classroom lectures on ticks. Teachers handed out the questionnaires, which were completed in the classroom, and they were returned to RIVM by mail.

This general survey among a sample of healthy children from the general population did not require formal medical ethical approval according to Dutch law [25].

### Analyses

We analyzed whether the game and the leaflet affect knowledge, perception and behavior in relation to ticks, tick bites and LB compared to the control group. Table 2 summarizes the design for this evaluation.

**Table 2:** Study design

	*t1* ^*a*^	*t2*
Game	*y*_*G*1_	*y*_*G*2_
Leaflet	*y*_*L*1_	*y*_*L*2_
Control	*y*_*C*1_	*y*_*C*2_

^a^Pre-intervention study [12]

*Y*_*G1*_ *and Y*_*G2*_ *=* intervention effect of game on t1 and t2, respectively

*Y*_*L1*_ *and Y*_*L2*_ *=* intervention effect of leaflet on t1 and t2, respectively

*Yc*_*1*_ *and Yc*_*2*_ *=* effect in control group on t1 and t2, respectively

Our main interest was any intervention effect for the game group, *y*_*G*2_ − *y*_*G*1_, and for the leaflet group, *y*_*L*2_ − *y*_*L*1_. Any observed difference *y*_*C*2_ − *y*_*C*1_ in the control group was used to determine whether there are learning effects and therefore mere measurement effects of only completing the questionnaires. In the presence of learning effects we considered a differences-in-differences (DID) design,^1^ by adjusting the differences *y*_*G*2_ − *y*_*G*1_ and *y*_*L*2_ − *y*_*L*1_for the difference *y*_*C*2_ − *y*_*C*1_. This gives us an estimate for the intervention effects that is adjusted for learning effects.

For our statistical analysis, we included only children whose school participated both at *t1* and *t2*. This selection of individuals makes it more plausible that any differences that may arise are the result of the difference in interventions, rather than any school-specific effects. From the pre-intervention study we concluded that substantial differences in knowledge of ticks exist between schools and therefore children [12].

Generalized linear mixed models (GLMM) were applied to analyze the intervention effects, whilst taking into account that the data are clustered [26]. Clusters are present in our data on two levels. First, children within the same class are likely to have a similar knowledge level, and therefore knowledge scores within classes are likely to be correlated. Second, the knowledge level of a child at *t2* depends on the initial knowledge level of this child at *t1*.

We chose to dichotomize our responses, and used a GLMM assuming a Bernoulli distribution and logit link (i.e., logistic regression with random effects).^2^ The knowledge level response was operationalized as a binary variable, by considering a minimum of 6 out of 7 questions correctly answered as sufficient, and insufficient otherwise (reference category).^3^ For the other responses, we defined the classes as follows: knowing other persons with Lyme - “yes” versus “no”/“don’t know” (reference), becoming ill after a tick bite (susceptibility) - “yes” versus “no”/“don’t know” (reference), importance of checking - “very”/“somewhat” versus “not important” (reference), and frequency of checks - “very often”/“sometimes” versus “not at all” (reference). For the questions related to knowing other persons with Lyme, and becoming ill after a tick bite, “no” and “don’t know” were pooled together because they both reflect, to varying degrees, the fact that respondents could not confirm the question. For the importance and frequency of checks, the categorization was chosen to see whether the intervention affected the proportion of individuals that take the risk of Lyme disease serious.

To address the sensitivity of our findings to this categorization, we also performed the analysis using an alternative categorization: becoming ill after a tick bite - “yes” versus “no” (leaving “don’t know” out), knowing other persons with Lyme - “yes” versus “no”, importance of checking - “very” versus “somewhat”/“not important”, and frequency of checks - “very often” versus “sometimes”/“not at all”. In most cases, we found that the results did not alter - with the exception of the check frequency, which will be discussed in the results below.

Three types of GLMM models were applied: (1) a model to estimate treatment effects in *t2* versus *t1*, (2) a model to estimate the difference in intervention effects between the game and leaflet groups, and (3) a model to estimate the intervention effects conditional on the covariate values (“knowing somebody with Lyme”, and “having had classroom lecture on ticks”). The rationale behind these models is as follows. In Model 1, we examined whether there exists any intervention effect at all for each group, without taking potential confounding into account. The assumption is made here that if a true effect exists, then this should already become apparent in this model because confounding over time (e.g., children might know more persons with Lyme disease in *t2* than in *t1*) within a group is relatively modest. And if such an effect is found, we applied Model 2 to determine whether this effect remained after adjusting for confounding over time, and whether this effect was sustained between groups after adjusting for differences in confounders between groups (e.g., one group might have had more classroom lectures on ticks than another). In particular, we were interested whether the game and leaflet groups performed better than the control group, and whether the game and leaflet groups performed differently from each other. Furthermore, based on an effect in Model 1, one might wonder whether this effect differs between subpopulations (e.g., the effect may be smaller for children who received classroom lectures on ticks), and if this difference exists, whether the difference varies by intervention group. To further describe these models, we introduce some mathematical notation. First, we define the intervention group *Z* as the group having been exposed to a specific intervention; *Z* can either be the game group, leaflet group or control group (i.e., no intervention at all). Let *Z*_*ij*_ be the group to which individual *i* in class *j* belongs. Furthermore, let *p*_*ij*_ be the probability of a success (e.g., a high knowledge score), *T*_*ij*_ the point of time and *x*_*ijk*_ the *k* ^*th*^ covariate respectively. *T*_*ij*_ = 0 refers to the baseline measurement, where *all* individuals are unexposed (because the leaflet was developed specifically for this study and not available anywhere). *T*_*ij*_ > 0 refers to the time points in which the game and leaflet groups become exposed to the intervention, but the control group remains unexposed. *T*_*ij*_ can be either 1 or 2. Note that we did not consider random effects on individual level in these models, for simplicity. It was not feasible to include models that take into account such random effects. Between-class variation was considered more important than between-person variation based on examination of the data.

#### Model 1: Treatment effects *t2* versus *t1*

$$ \log \left(\frac{p_{ij}}{1-{p}_{ij}}\right)=\left({\alpha}_0+{a}_{0j}\right)+\left({\alpha}_1+{a}_{1j}\right){T}_{ij}, $$

Where *a*_0*j*_ and *a*_1*j*_ are a random intercept and random slope for classes, respectively. *α*_1_ provides the intervention effect for an individual (which can be interpreted as changes in *p*_*ij*_ on a logit scale per individual between *t2* and *t1*). This model was fitted separately per intervention group *Z*, and for all outcomes.

#### Model 2: Differences in intervention effects between intervention groups

$$ \log \left(\frac{p_{ij}}{1-{p}_{ij}}\right)=\left({\beta}_0+{b}_{0j}\right)+\left({\beta}_1+{b}_{1j}\right){T}_{ij}+{\beta}_2{Z}_{ij}+{\beta}_3{G}_{ij}{T}_{ij}+{\displaystyle \sum_k{\beta}_k}{x}_{ijk}+{\displaystyle \sum_k{\beta}_{k+m}}{x}_{ijk}{T}_{ij} $$

Here, *β*_1_ provides the intervention effect for the reference group (game group), *β*_2_ the difference between intervention groups at baseline, and *β*_3_ the difference in intervention effects between intervention groups at *t*. Here, we are mainly interested in *β*_3_. *β*_*k*_ and *β*_*k* + *m*_represent the influence of confounding covariate *k*, but are not of interest. This model was fitted across all intervention groups. This model was considered for all outcomes. Covariates used were “knowing somebody with Lyme”, and “having followed classes on tick bites”.

#### Model 3: Conditional intervention effects

$$ \log \left(\frac{p_{ij}}{1-{p}_{ij}}\right)=\left({\gamma}_0+{c}_{0j}\right)+\left({\gamma}_1+{\gamma}_{ij}\right){T}_{ij}+{\displaystyle \sum_k{\gamma}_k}{x}_{ijk}+{\displaystyle \sum_k{\gamma}_{k+m}}{x}_{ijk}{T}_{ij} $$

Here, *γ*_*k* + *m*_, gives the intervention effect conditional on *x*_*k*_. This model is fitted separately per intervention group *Z*. This model was considered for all outcomes. Covariates that were considered are “knowing somebody with Lyme”, and “having had classroom lecture on ticks”.

## Results

Table 3 shows the descriptive statistics for each outcome variable at *t1* and *t2*. At *t1*, most respondents in all groups find tick checks important, ranging from 90.9 to 94.5%. All respondents had a high knowledge score (≥6/7 knowledge items correct), ranging from 69.2 to 72.3%. The differences across groups at *t1* are relatively small, with the exception of the percentage of respondents checking for tick bites; this ranged from 55.1% in the game group to 76.4% in the control group. The percentages are higher across nearly all outcome variables (depicted in the last three columns of Table 3), although the increase for the knowledge score is substantially higher (16.6 to 23.3% across all intervention groups). Using Model 1, we tested whether these treatment effects were significant. The respondents in the two intervention groups and the control group had a significant better knowledge (≥6/7 questions answered correctly) at *t2* than at *t1* (p < 0.001). Three questions seem to be relatively difficult, namely estimating tick size (78.3 to 84.7% correct), knowing the location of ticks in vegetation (61.6 to 72.8%), and knowing where ticks bite (71.3 to 79.6%) (Additional file 3: Appendix 3). Unsurprisingly, these items showed the biggest intervention effects, since these are also the items where the largest gain could be achieved. These differences were found to be statistically significant (based on Model 1). Also statistically significant were improvements in knowledge for estimation of tick size in the leaflet group (12.9%), and knowing where on the body ticks bite for the game and leaflet group (12.1 and 17.1%, respectively).

**Table 3:** Descriptive statistics and effects per intervention group over time, based on Model 1

	*t1*			*t2*			Difference *t2* – *t1*		
	Game (*n* = 254)	Leaflet (*n* = 328)	Control (*n* = 399)	Game (*n* = 199)	Leaflet (*n* = 316)	Control (*n* = 372)	Game (*n* = 199)	Leaflet (*n* = 316)	Control (*n* = 372)
% of respondents with a knowledge score ≥6/7 (knowledge items correct)	70.0	72.3	69.2	88.0	95.6	85.8	**18.0** ***p < 0.001***	**23.3** ***p < 0.001***	**16.6** ***p < 0.001***
% of respondents that perceived that they are personally susceptible for illness after a tick bite (1st item for perceived susceptibility)	68.1	67.7	71.0	78.9	71.1	60.4	10.8 *p = 0.08*	3.4 *p = 0.29*	−10.6 *p = 0.05*
% of respondents that personally knows someone with illness after tick bite (2nd item for perceived susceptibility)	20.2	26.2	30.0	20.3	33.5	30.2	0.1 *p = 0.97*	7.3 *p = 0.18*	0.2 *p = 0.81*
% of respondents that regards tick checks as ‘somewhat’ or ‘very important’	90.9	91.5	94.5	94.9	91.8	96.1	4.0 *p = 0.35*	0.3 *p = 0.77*	1.6 *p = 0.66*
% of respondents checked for tick bites occasionally or every time after playing in green areas	55.1	67.1	76.4	68.5	67.3	82.7	**13.4** ***p = 0.02***	0.2 *p = 0.98*	6.3 *p = 0.24*

In bold: statistically significant values

Furthermore, the frequency of reported tick checks increased significantly in the game group (*p* = 0.02). In sensitivity analysis (using ‘very often checking’ versus ‘no/sometimes checking’ definition) this effect was no longer significant. Other outcomes were not analyzed further in Model 2 and 3, since in Model 1 no other intervention effects were identified.

To identify any differences in intervention effects between the groups, we estimated Model 2 (Table 4). This was only done for the knowledge and the tick-check frequency outcomes, since there were no significant effects found for the outcomes perceived susceptibility and tick-check importance (Table 3). In Model 2 we adjusted the knowledge outcome (based on sum score) and the tick check frequency for the confounders “knowing somebody with Lyme” and “having had a classroom lecture on ticks”. The parameter estimates that are of interest are *t2* and the interactions t2*leaflet and t2*control.

**Table 4:** Differences in intervention effects on knowledge and tick check frequency between intervention groups and control group after adjusting for confounders (knowing somebody with Lyme and having had lectures on ticks), based on Model 2

	**Knowledge**			**Tick check frequency**		
	**β**	**S.E.**	***p*** **value**	**β** ^**a**^	**S.E.**	***p*** **value**
(Intercept)	0.522	0.265	**0.049**	−0.034	0.241	0.886
*t2* (reference: *t1*)	1.430	0.310	**0.000**	0.599	0.252	**0.017**
Leaflet (reference: game)	0.232	0.337	0.499	0.624	0.305	0.052
Control group (reference: game)	0.032	0.330	0.924	1.040	0.305	**0.002**
Knowing somebody with Lyme	0.604	0.178	**0.001**	0.710	0.178	**0.000**
Having had lectures on ticks	0.535	0.184	**0.004**	0.215	0.176	0.222
*t2* ^a^Leaflet	0.735	0.409	0.072	−0.601	0.291	**0.039**
*t2* ^a^Control	−0.532	0.335	0.112	−0.347	0.298	0.244
*t2* ^a^Knowing somebody with Lyme	−0.022	0.316	0.946	−0.270	0.253	0.286
*t2* ^a^Having had lectures on ticks	−0.380	0.289	0.188	0.035	0.241	0.883

In bold: statistically significant values

^a^Beta refers to the regression coefficient from the GLMM model. If for a given covariate Beta is greater (smaller) than zero, and the corresponding *p*-value is significant, then the covariate is positively (negatively) associated with the outcome (either knowledge or tick check frequency). If the Beta is not significant, then no evidence of an association between covariate and outcome was found

For knowledge (based on sum score) the main effect *t2* is significant but the interaction t2*control is not significant, which suggests that the increase in knowledge purely comes from a learning effect. In (Additional file 4: Appendix 4) these interactions are presented for the three knowledge questions that demonstrated the biggest effects. The interaction t2*control is positive and significant for preferred location in vegetation and bite site on body, suggesting that the game intervention increased participants’ knowledge for these two items more compared to the control group. This interaction is not significant for tick size, which suggests that the increase in knowledge on tick size purely comes from the aforementioned learning effect.

The interaction t2*leaflet is only positive and significant for the tick size outcome, which suggests that the leaflet performs better than the game in terms of informing the respondents on that aspect. Since t2*control is not significant for this outcome, it seems that the leaflet group performed better than both the game and control group.

For tick-check frequency, the interaction t2*leaflet is positive and significant (Table 4), suggesting the game improved the tick-check frequency more than the leaflet. The interaction t2*control is not significant, however, which suggests that the increase in tick-check frequency is a learning effect.

Finally, in a third model we analyzed whether the intervention effects on knowledge and on tick-check frequency might be influenced by the confounders “Knowing somebody with Lyme” and “Having had classroom lecture on ticks” [12]. These were not significant for all outcome variables, suggesting that they may only affect knowledge level and tick-check frequency in general, but not the effects of the interventions (Additional file 5: Appendix 5 and Additional file 6: Appendix 6)

## Discussion

We can conclude from the results of this study that both the game and the leaflet interventions had a positive effect on the children in terms of improving the prevention of tick bites and LB. This is in line with a recent study of Shadick et al. who showed that a short in-class LD education program based on social learning theory impacted a child's knowledge, attitude, and preventive behavior [27].

Model 1 showed the effects of the interventions. Although the knowledge level of the children was already high before, at the pre-intervention measurement (*t1*), overall knowledge improved significantly for all three groups (i.e., game, leaflet, control). In addition, the frequency of checks for ticks increased significantly in the game group.

In terms of knowledge improvement, it appears the leaflet performs better than the game in informing children about the typical size of ticks. This can probably be explained by the presentation of the tick size: in the game, the tick was pictured as a humorous illustration of a small black spider, while in the leaflet it was an image of a tick drawn to real size. In retrospect, we realized that the children in the game group were therefore informed incorrectly about the size of a tick. This is a pitfall of developing computer games (for children); it has to be educational and veracious, but at the same time it has to be sufficiently attractive and entertaining to be able to compete with other games and retain the player’s attention to complete the game. As Thompson stated in his paper about the role of educational video games in obesity prevention: “this game genre has the formidable task of achieving a balance between “fun-ness” (i.e., components that entertain, such as animation, storyline, sound effects) and “serious-ness” (i.e., the components that promote behavior change, such as goal setting, problem solving)” [28]. This is linked to the current debate on how enjoyable games for health should be in order to be effective. On the one hand, it has been argued that health games (or, more broadly, educational games) will not be effective if they provide insufficient enjoyment; precisely because of this, fundamentally they do not differ from entertainment games in terms of game type, design, or dissemination strategy. On the other hand, it has been argued that educational games should not compete with entertainment games in terms of enjoyment, but instead with its analogous alternative (e.g., school curricula, behavior change interventions delivered in other settings) [29]. In the future the game can be improved by using an image of a tick drawn to real size.

In terms of tick checks, it appeared that at the pre-intervention measurement (*t1*), only 18% of the children were routinely checked by their parents after potential exposure to ticks [12]. In the game group the frequency of tick checks increased significantly; the percentage of respondents that checked or were checked for tick bites occasionally or every time after playing in green areas increased from 55.1 to 68.5% (13.4%, *p = 0.02*). Since the percentage of respondents checking for tick bites was lowest in the game group, there also the largest gain could be achieved. In the sensitivity analysis, using the definitions “checking very often” versus “no checking/checking sometimes”, this effect was no longer statistically significant. This suggests that the game had a clear effect on the children who did not check before the game, but did not influence (positively nor negatively) those who already checked sometimes or frequently.

Model 2 showed the differences in intervention effects between the intervention groups and the control group after adjustment for the confounders “knowing somebody with Lyme” and “having had lectures on ticks”. With this model we could not demonstrate significant differences in knowledge improvement between the game, the leaflet or the control groups. The game was better than the leaflet in improving preventive behavior, but no better than the control. These positive effects observed in the control group suggest the existence of a ‘learning effect’ of completing the questionnaire. Children may improve their knowledge and behavior by simply completing the questionnaire, and possible looking up the correct answers afterwards (by looking on the internet, or discussing the questions in the classroom with the teacher and/or other children or their parents). Evidence indicates that receiving a questionnaire about a behavior increases the likelihood that the person will perform that behavior. This is known as the mere measurement effect [30] and suggests that even a simple questionnaire can result in changes in knowledge and preventive behavior (frequency of ticks checks). In response to this effect, we think it would be a promising idea to add a ‘ticks and Lyme quiz’ to the available tools for public health education (in schools).

Papastergiou concluded in her literature review about the potential of computer and video games for health education that computer gaming is more appealing than, and at least as effective as, conventional instructional media in positively influencing health-related knowledge, attitudes and behaviors. She suggested that this perhaps indicates that games should be viewed as a complement to other media within health education programs [18].

This study has some noteworthy restrictions. It should be noted that only children attending the two final years (grade 7 and 8) of primary school participated in this study. Thus, we can only derive conclusions for the effect of the online educational game in the age range 9–13 years. Future research could investigate the potential effects of the game on children of other age groups.

A limitation of the study at hand is that the baseline measurement (t1) was conducted two months before participants were exposed to the actual interventions. Changes might have happened in this period between measurement and exposure. However, we do not expect these changes to affect differences between groups, as only after this period participants were randomized to the different intervention groups. Nevertheless, in future studies it would be warranted to conduct baseline measurement just before actual exposure to the intervention. In this study, schoolchildren were allocated randomly to the game, leaflet or control groups. In real life, children can often decide themselves which educational tool they choose. Some children like to play a game, while others may prefer to read a leaflet. We asked the children what they feel the preferred method for education on tick bites should be. In all groups, a video game was most popular, followed by explanation by a teacher. Therefore, it is feasible that some of the children played the game or read the leaflet against their preference. This might have reduced the effects of the tools. This fits in with the conclusion of Schulz et al. that the best kind of intervention may depend on personal preferences, so called ‘preference-based tailoring’ [31].

Finally, the intervention in this study was aimed at children and therefore we asked the children, and not the parents, pre-intervention and post-intervention whether their parents performed a tick check. A survey of the parents of the participants could potentially have enriched the study, to determine the influence of the children on their parents’ knowledge, perception and preventive behavior regarding ticks and tick bites.

## Conclusion

This is the first online educational video game on ticks and tick bites. The game is effective in improving knowledge and preventive behavior regarding ticks and tick bites. However, it did not outperform the leaflet or control groups on all outcome measures. The positive effects observed in the control group suggest the existence of a mere measurement effect related to completion of the study questionnaire. Nevertheless, the educational video game can play a complementary role to other media within the health education program on ticks and LD aimed at children.

## Additional files

Additional file 1: Appendix 1 Questionnaire. (DOCX 16 kb)
Additional file 2: Appendix 2. (DOCX 516 kb)
Additional file 3: Appendix 3. (DOCX 15 kb)
Additional file 4: Appendix 4. (DOCX 17 kb)
Additional file 5: Appendix 5. (DOCX 15 kb)
Additional file 6: Appendix 6. (DOCX 15 kb)

## Footnotes

1 This is not a ‘traditional’ DID design in the sense that individual children could not be identified. Rather, schools were identifiable, so the DID design was applicable on a grouped level (i.e., schools).

2 This was done for simplification purposes. The statistical software R was used in this study. We were not aware of any (non-Bayesian) package supporting the modelling of a response with three or more classes, whilst taking into account multiple random effects.

3 The reason why this bar was set so high was that children scored that high at the knowledge level in the study at t1 [Beaujean 2013].
