# Supplementary material for: Education on tick bite and Lyme borreliosis prevention, aimed at schoolchildren in the Netherlands: comparing the effects of an online educational video game versus a leaflet or no intervention
Source: BMC Public Health. 2016 Nov 16;16:1163. doi: 10.1186/s12889-016-3811-5 (PMC5112636; doi:10.1186/s12889-016-3811-5)
Supplement: Additional file 1: — Appendix 1 Questionnaire. (DOCX 16 kb) [file 12889_2016_3811_MOESM1_ESM.docx]

**Appendix 1 Questionnaire**

In the pre-intervention study at t1, questions 1-13 were asked.

At t2, questions 1-24 were asked in the game group, 1-23 in the leaflet group, and questions 1-13 and 22 in the control group.

What is your first name? ………………….

Girl / boy

What age are you? ………………………..

In which class are you? …………………..

1) Did you ever receive classroom lectures about ticks?

a. Yes

b. No

2) What does a tick look like?

a. Image: Silhouette of an ant

b. Image: Silhouette of a tick

c. I don’t know

3) What is the real size of a tick that could bite you?

a. Image: poppy-seed size black dot

b. Image: pea size black dot

c. I don’t know

4) At what sort of place would you expect to encounter ticks?

a. Color image of path in a forest

b. Color image of sand-covered playground

c. I don’t know

5) Why should you watch out for ticks?

a. Ticks make you itch

b. Ticks can make you ill

c. I don’t know

6) At what place do ticks prefer to live?

a. Up in the trees

b. Near the ground in brushes and tall grass

c. I don’t know

7) What can you do to prevent tick bites?

a. Wash carefully to rinse off ticks

b. Check your clothes and body with an adult and pull out attached ticks

c. I don’t know

8) Which picture shows best where ticks prefer to bite?

a. Image: child body contours with arrows pointing at nose, chest, hands and toes

b. Image: child body contours with a rows pointing at ears, armpits crotch and knees

c. I don’t know

9) Do you think that you could personally become ill after a tick bite?

a. Yes

b. No

c. I don’t know

10) Do you consider it important to be checked for tick bites after playing in an area where ticks may live?

a. Not important

b. Somewhat important

c. Very important

11) Do you know someone who has become ill after a tick bite?

a. Yes

b. No

c. I don’t know

12) Did you ever do a body inspection for tick bites with your parent or carer?

a. Never

b. Occasionally

c. Every time after a visit to an area where ticks may live

13) Did you ever had a tick bite?

a. Never

b. Once

c. More than once

14) How often did you play the game Teek control?

a. Never

b. Once

c. More than once

15) Did you like the game / leaflet?

a. The game is not amusing

b. The game is a bit of amusing

c. Yes, the game is amusing

16) Is the game / leaflet difficult to play/read?

a. Difficult

b. Bit difficult, bit easy

c. Easy

17) Does it take long time to play the game / read the leaflet?

a. Too long

b. Just long enough

c. Too short

18) Did you sent the e-mail to your parents/carers at the end of the game?

a. Yes

b. No

c. I don’t know

19) Did you send the game to your friends or family?

a. Yes

b. No

c. I don’t know

20) If you have send it, how did you do that? (multiple answers possible)

a. Via E-mail

b. Via Hyves

c. Via Facebook

d. Via Twitter

e. Via conversation

21) Do you like to play the game / read the leafet (again), for example at home?

a. Yes

b. No

c. I don’t know

22) How do you prefer to learn about ticks?

a. Through game

b. Through teacher at school

c. Through leaflet

d. Through parents/carers

23) What did you learn from the game Teek control / leaflet?

…………………………………………………………………………………

…………………………………………………………………………………

…………………………………………………………………………………

24) Give the game a score between 0 (very bad) and 10 (very good)

………………..
