# Supplementary figures and images for: Education on tick bite and Lyme borreliosis prevention, aimed at schoolchildren in the Netherlands: comparing the effects of an online educational video game versus a leaflet or no intervention
Source: BMC Public Health. 2016 Nov 16;16:1163. doi: 10.1186/s12889-016-3811-5 (PMC5112636; doi:10.1186/s12889-016-3811-5)

**Additional file 2: Appendix 2. Leaflet**


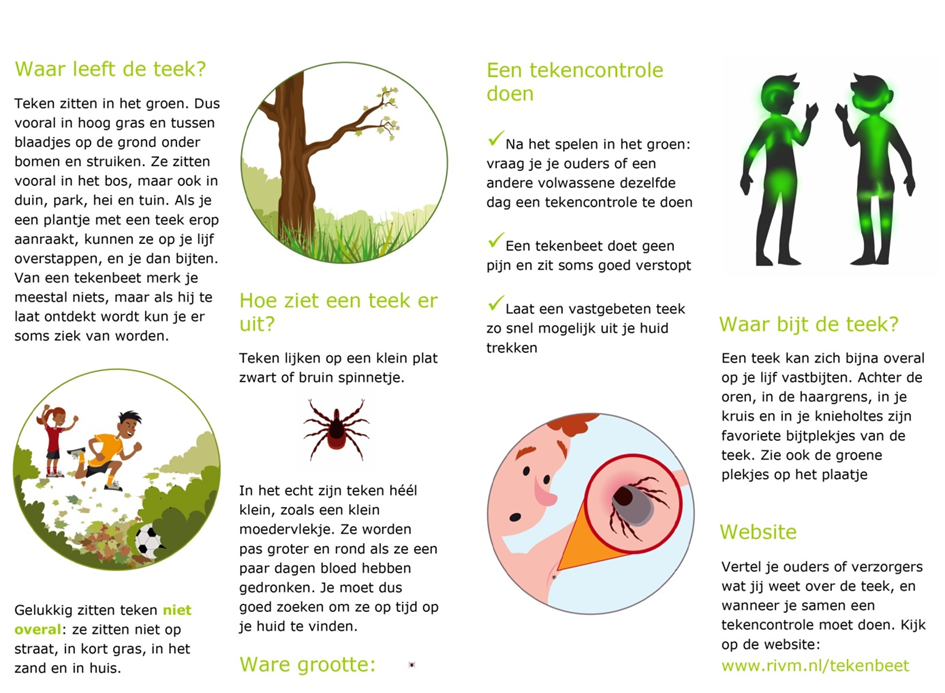

Supplement: Additional file 2: — Appendix 2. (DOCX 516 kb) [file 12889_2016_3811_MOESM2_ESM.docx]
