# Supplementary material for: Education on tick bite and Lyme borreliosis prevention, aimed at schoolchildren in the Netherlands: comparing the effects of an online educational video game versus a leaflet or no intervention
Source: BMC Public Health. 2016 Nov 16;16:1163. doi: 10.1186/s12889-016-3811-5 (PMC5112636; doi:10.1186/s12889-016-3811-5)
Supplement: Additional file 3: — Appendix 3. (DOCX 15 kb) [file 12889_2016_3811_MOESM3_ESM.docx]

**Additional file 3: Appendix 3. Table 3**

|  | ***t1*** | | | ***t2*** | | | **Difference *t2* – *t1*** | | |
| --- | --- | --- | --- | --- | --- | --- | --- | --- | --- |
|  | Game | Leaflet | Control | Game | Leaflet | Control | Game | Leaflet | Control |
| **Knowledge item** | (n = 254) | (n = 328) | (n = 399) | (n = 199) | (n = 316) | (n = 372) | (n = 199) | (n = 316) | (n = 372) |
| Recognising a tick (% correct) | 88.6 | 89.3 | 83.4 | 98.5 | 98.4 | 93.0 | 9.9 | 9.1 | 9.6 |
| Estimating tick size (% correct) | 78.3 | 84.2 | 84.7 | 83.4 | 97.1 | 90.8 | 5.1 | **12.9** | 6.1 |
| Recognising a typical tick area (% correct) | 96.8 | 99.1 | 98.7 | 99.5 | 99.7 | 99.7 | 2.7 | 0.6 | 1.0 |
| Being aware of potential tick bite consequence (% correct) | 93.7 | 93.0 | 94.5 | 98.0 | 99.7 | 98.7 | 4.3 | 6.7 | 4.2 |
| Knowing tick position in vegetation (% correct) | 72.8 | 65.5 | 61.6 | 91.0 | 91.8 | 68.3 | **18.2** | **26.3** | 6.7 |
| Knowing how to remove ticks (% correct) | 86.9 | 85.4 | 83.5 | 96.4 | 96.5 | 94.3 | 9.5 | 11.1 | 10.8 |
| Knowing where on the body ticks bite (% correct) | 71.3 | 76.8 | 79.6 | 83.4 | 93.9 | 81.2 | **12.1** | **17.1** | 1.6 |

In bold statistically significant differences.

**Table 3 Descriptive statistics for 7 knowledge questions per intervention at *t1* and *t2***
