# Supplementary material for: Education on tick bite and Lyme borreliosis prevention, aimed at schoolchildren in the Netherlands: comparing the effects of an online educational video game versus a leaflet or no intervention
Source: BMC Public Health. 2016 Nov 16;16:1163. doi: 10.1186/s12889-016-3811-5 (PMC5112636; doi:10.1186/s12889-016-3811-5)
Supplement: Additional file 4: — Appendix 4. (DOCX 17 kb) [file 12889_2016_3811_MOESM4_ESM.docx]

**Appendix 4
Comparison of differences in intervention effects for the three knowledge questions with the biggest effects, after adjusting for confounders (knowing somebody with Lyme and having had lectures on ticks), based on Model 2**

|  |  |  | |  |  |  |  | |  |  | | | |  |  |  | |  |
| --- | --- | --- | --- | --- | --- | --- | --- | --- | --- | --- | --- | --- | --- | --- | --- | --- | --- | --- |
|  |  | **Tick size** | | |  |  | **Preferred location** | | | **Preferred bite site** | | | |  |  |  |  |  |
|  |  | **Value** | **S.E.** | | **p value** |  | **Value** | **S.E.** | **p value** |  | **Value** | **S.E.** | **p value** | | | |  |  |
| (Intercept) |  | 1.050 | 0.275 | | **0.000** |  | 0.709 | 0.235 | **0.003** |  | 0.626 | 0.309 | **0.043** | | | |  |  |
| t2 (reference: t1) |  | 0.562 | 0.306 | | 0.066 |  | 1.570 | 0.319 | **0.000** |  | 1.130 | 0.290 | **0.000** | | | |  |  |
| Leaflet (reference: game) |  | 0.508 | 0.357 | | 0.168 |  | -0.227 | 0.296 | 0.451 |  | 0.421 | 0.393 | 0.295 | | | |  |  |
| Control group (reference: game) |  | 0.578 | 0.352 | | 0.114 |  | -0.321 | 0.290 | 0.280 |  | 0.551 | 0.389 | 0.171 | | | |  |  |
| Knowing somebody with Lyme |  | 0.248 | 0.212 | | 0.244 |  | 0.394 | 0.165 | **0.017** |  | 0.480 | 0.197 | **0.015** | | | |  |  |
| Having had lectures on ticks |  | 0.445 | 0.228 | | 0.051 |  | 0.111 | 0.171 | 0.515 |  | 0.675 | 0.215 | **0.002** | | | |  |  |
| t2*Leaflet |  | 1.310 | 0.443 | | **0.003** |  | 0.334 | 0.375 | 0.373 |  | 0.514 | 0.372 | 0.167 | | | |  |  |
| t2*Control |  | 0.063 | 0.345 | | 0.855 |  | -1.160 | 0.331 | **0.000** |  | -0.929 | 0.318 | **0.004** | | | |  |  |
| t2*Knowing somebody with Lyme |  | 0.473 | 0.387 | | 0.221 |  | -0.221 | 0.258 | 0.392 |  | -0.143 | 0.305 | 0.639 | | | |  |  |
| t2*Having had lectures on ticks |  | -0.567 | 0.341 | | 0.096 |  | -0.186 | 0.253 | 0.462 |  | -0.623 | 0.299 | **0.037** | | | |  |  |

In bold: statistically significant values p<0.05.
