# Supplementary material for: Education on tick bite and Lyme borreliosis prevention, aimed at schoolchildren in the Netherlands: comparing the effects of an online educational video game versus a leaflet or no intervention
Source: BMC Public Health. 2016 Nov 16;16:1163. doi: 10.1186/s12889-016-3811-5 (PMC5112636; doi:10.1186/s12889-016-3811-5)
Supplement: Additional file 5: — Appendix 5. (DOCX 15 kb) [file 12889_2016_3811_MOESM5_ESM.docx]

**Appendix 5**

**Conditional treatment effects on knowledge, after adjusting for confounders (knowing somebody with Lyme and having had lectures on ticks), based on Model 3**

|  |  |  |  |  |  |  |  |  |  |
| --- | --- | --- | --- | --- | --- | --- | --- | --- | --- |
|  | **Game** | | | **Leaflet** | | | **Control** | | |
|  | **Value** | **S.E.** | **p-value** | **Value** | **S.E.** | **p value** | **Value** | **S.E.** | **p value** |
| (Intercept) | 0.461 | 0.383 | 0.228 | 0.883 | 0.238 | **0.000** | 0.498 | 0.166 | **0.003** |
| t2-t1 | 1.560 | 0.430 | **0.000** | 2.330 | 0.431 | **0.000** | 0.887 | 0.259 | **0.001** |
| Knowing somebody with Lyme | 0.588 | 0.389 | 0.132 | 0.468 | 0.296 | 0.115 | 0.736 | 0.264 | **0.005** |
| Having had classroom lecture on ticks | 0.844 | 0.382 | **0.028** | 0.098 | 0.300 | 0.745 | 0.575 | 0.285 | **0.044** |
| t2-t1:Knowing somebody with Lyme | 0.022 | 0.751 | 0.976 | 0.229 | 0.695 | 0.742 | -0.143 | 0.422 | 0.735 |
| t2-t1:Clasrooms lectures on ticks | -0.885 | 0.605 | 0.144 | -0.424 | 0.604 | 0.483 | -0.196 | 0.412 | 0.634 |

In bold: statistically significant values p<0.05.
