# Supplementary material for: Education on tick bite and Lyme borreliosis prevention, aimed at schoolchildren in the Netherlands: comparing the effects of an online educational video game versus a leaflet or no intervention
Source: BMC Public Health. 2016 Nov 16;16:1163. doi: 10.1186/s12889-016-3811-5 (PMC5112636; doi:10.1186/s12889-016-3811-5)
Supplement: Additional file 6: — Appendix 6. (DOCX 15 kb) [file 12889_2016_3811_MOESM6_ESM.docx]

**Appendix 6**

**Conditional treatment effects on tick check frequency, after adjusting for confounders (knowing somebody with Lyme and having had lectures on ticks), based on Model 3**

|  |  |  |  |  |  | |  |  |  |  |
| --- | --- | --- | --- | --- | --- | --- | --- | --- | --- | --- |
|  | **Game** | | | **Leaflet** | | | | **Control** | | |
|  | **Value** | **S.E.** | **p value** | **Value** | **S.E.** | **p value** | | **Value** | **S.E.** | **p value** |
| (Intercept) | 0.461 | 0.382 | 0.228 | 0.886 | 0.238 | **0.002** | | 0.498 | 0.166 | **0.003** |
| t2-t1 | 1.555 | 0.433 | **0.000** | 2.340 | 0.433 | **0.000** | | 0.887 | 0.259 | **0.001** |
| Knowing somebody with Lyme | 0.588 | 0.389 | 0.131 | 0.466 | 0.296 | 0.115 | | 0.736 | 0.264 | **0.005** |
| Having had classroom lecture on ticks | 0.844 | 0.381 | **0.027** | 0.094 | 0.299 | 0.753 | | 0.575 | 0.285 | **0.044** |
| t2-t1:Knowing somebody with Lyme | 0.035 | 0.750 | 0.962 | 0.225 | 0.695 | 0.746 | | -0.143 | 0.422 | 0.735 |
| t2-t1:Clasrooms lectures on ticks | -0.881 | 0.605 | 0.146 | -0.464 | 0.604 | 0.443 | | -0.196 | 0.412 | 0.634 |

In bold: statistically significant values p<0.05.
